# Periscope

**Published:** 1889-09

**Authors:** 


### PERISCOPE.

### MEDICINE.

**Mode of Infection in Diphtheria.**—Dr. Sevestre believes that though diphtheria can be transmitted by the atmosphere, yet contagion is most frequently carried from the infected to the healthy person by means of some intermediate object. He gives two instances in which this mode of infection clearly appeared to have taken place:

In the first, a patient suffering from typhoid fever was visited by his sister, a nurse in the diphtheria wards in the Hospital Trousseau; she left with him a shawl that she was in the habit of wearing in the wards, and some days afterwards he was taken with diphtheria.

The second instance was that of a young girl, whose mother had died of diphtheria two years before: there was no possibility of her having been in contact with a case of diphtheria since then, and she lived under good sanitary conditions. Various things that the mother had used during her last illness were locked up in a chest. Some days before she herself contracted diphtheria, the child had been pulling over these things, which had been undisturbed for two years, with her sister. In this case there seemed to be no possible source of infection except through these articles, that had therefore retained the virus unimpaired for two years.

Dr. Sevestre accordingly strongly insists on the necessity of thoroughly disinfecting or destroying everything used by a diphtheritic patient.—*Le Progrès médical*, March 2nd, 1889.

J. M. C.

**Equino-telluric origin of Tetanus.**—M. Verneuil at the French Academy of Medicine embodied the results of his observations on this subject in the following conclusions:

(1) Tetanus is transmitted not only from one animal to another of the same or of different species, but also from man to man, from men to animals and *vice versâ*.

(2) Contagion from a horse suffering from tetanus to a man with an open wound is brought about directly or indirectly; most frequently in the latter way.

(3) By means of intermediate agents. These comprise all objects, of whatever kind which, from brief or prolonged contact with a horse affected with tetanus, receive the virus without destroying it.

(4) Living beings may carry the infection without being themselves affected by it; but the presence of an open wound at once renders them liable to auto-inoculation.

(5) A man with an open wound may be infected by any object that carries the virus; but observation and experiment show that the most dangerous source of contagion is derived from the horse and his surroundings; next dangerous is contact with cultivated soil, which only certainly possesses the power of causing tetanus in man when it has been contaminated by a horse affected with the disease.

(6) Statistics show that, as in the case of glanders, those who have the care of horses are the chief sufferers from tetanus: the topographical distribution of equine and human tetanus coincide. Equine is more common than human tetanus in our climate. When equine tetanus diminishes and tends to disappear, human does so in corresponding proportion. The prophylaxis of tetanus lies therefore in veterinary hygiene.—*Le Progrès médical*, April 6th, 1889.

J. M. C.

**Obesity.**—Kisch, in a recent monograph on this subject, gives an account of an analysis of 10,000 cases. In 100 cases which were examined as to the state of the hæmoglobin, it was increased in 79 and decreased in 21. The mode of the occurrence of fatty infiltration of the heart was studied experimentally on geese and seen to result in the later stages in a partial degeneration of the muscular fibres from pressure by fatty accumulation. This accounts for the disturbance of function, pressure on the ganglion-cells lying in the sulci probably causing it in the earlier phases. Sphygmographic tracings show that out of 700 cases only 20 were normal—2.8 per cent.: in 43.5 per cent. there was a slow pulse of different degrees, while a dicrotic pulse was seen in 32.2 per cent. There was increased tension, through arterio-sclerosis, in 16.1 per cent., and irregularity in 8 per cent. He considers the slow pulse an important symptom of fatty infiltrated heart. He found that often where there was a considerable slowing of the pulse to the extent of 50—30 beats, where there was a great amount of distress with insufficient objective condition to account for it, sudden death often occurred. In the liver he held that there is a retention of bile and formation of gallstones; in contradistinction to Murchison, who denied the tendency to jaundice. In 38 autopsies, gallstones were found in three. Albuminuria occurred frequently—in moderate amount and transitory, where no appearances pointed to affection of the epicardial fat; in greater amount and lasting, where due to passive congestion and slight parenchymatous nephritis. Transitory glycosuria, passing in the course of years into diabetes mellitus, is said to occur often. Menstrual anomalies are seen frequently. In 215 women, 48 were sterile—21 per cent.—Review in *Berliner klinische Wochenschrift*, No. 36, 1888. D. R. P.

**The Functions of the Stomach in Phthisis.**—At the recent Medical Congress held at Wiesbaden, Professor Immermann of Basel discussed the question whether the anorexia observed in cases of phthisis was merely a form of digestive hyperæsthesia or due to an actual disturbance of the gastric function. To settle this point, experiments were instituted with the test-meal of Leube and Riegel. Cases were chosen where the appetite was still good, as well as cases where there was disinclination for food; and also patients where the pulmonary affection was very advanced, especially those where high fever was present. In all cases the test-meal was digested in six hours, even in those where the appetite failed, where dyspeptic feelings were present, the strength poor and the fever very high. In one case, even five days before death, the meal was well digested. The probability therefore is that these gastric symptoms have a subjective or nervous character. The degree of acidity present one hour after the ingestion of a meal was the same as in the healthy condition, while the amount of pepsin showed its normal proportion. Immermann concludes from this that we ought to be more confident in the administration of food to phthisical patients than we usually are.—*Berliner klinische Wochenschrift*, No. 18, 1889. D. R. P.

### THERAPEUTICS.

**Treatment of Tænia.**—M. Descroizilles prefers the ethereal extract of male fern. For six weeks before the remedy is administered the patient abstains from sugar, pastry, &c., taking a gentle purgative every fifteen days; for the last four days milk-diet only is allowed. At night, and on the following morning at 5 a.m., an enema is given, then the male fern is taken at 8 a.m. The child stays in bed to prevent vomiting, and two hours after takes a dose of castor oil. If an evacuation of the bowels does not shortly follow, an enema is given. If a cure does not result, one should wait three months before making a second attempt.

M. Béranger-Ferand allows the patient only bread and milk for his supper the evening before taking the vermifuge:

at 6 a.m. a senna draught is taken, followed by the vermifuge at 6.30, and a second aperient dose one hour later.

He has tried cucurbita maxima, kamala, and pelletierine. The two former give rise to nausea, sometimes vomiting, colicky pains, headache, and are not effectual. Kamala causes the expulsion of live fragments of the worm, but does not cure, as the head remains. Pelletierine (dose for adult 3—6 grains) gave the best results; it was successful in 72 per cent. of cases. Dujardin-Beaumetz says that pelletierine stupefies the tapeworm for a very short time, but does not expel it; a quickly-acting purgative should be given about twenty minutes after it. He adds a caution against the use of pelletierine in the case of young children. Tannin, when given with pelletierine, renders it insoluble, so that it is not absorbed by the stomach. The only contra-indication against pelletierine for adults is that it gives rise to vomiting, which may perhaps be avoided by previously giving cocaine or antipyrin. —*L'Union médicale*, April 30th, 1889. J. M. C.

**Treatment of Ileus.**—In the discussion upon this subject at the Wiesbaden Congress, Curschmann, who opened the question, stated that out of 105 cases of serious intestinal obstruction, 37, or more than a third, recovered spontaneously. He looks upon the difference of opinion in the matter of treatment between physicians and surgeons as due to the fact that the latter generally see cases where the obstruction is complete, and the patient is in a serious condition. Fürbringer declared that one-third of the cases admitted into the Friedrichhain Hospital recovered without operation. It might be said from the evidence of statistics that the result of internal treatment is not bad enough, and that of surgical treatment not sufficiently good, to necessitate calling in the aid of the surgeon. Excellent results are obtained from opium. Puncture of the intestine is not dangerous, and it has led in many cases to improvement. The injection of air into the intestine has good effect, but in some cases disagreeable consequences follow. Von Ziemssen supported this latter treatment, and stated that during it one can often by auscultation determine the seat of obstruction. Fæcal vomiting may occur from a communication between the stomach and colon, and this can be recognised by pouring water into the stomach and injecting air into the rectum. Ziemssen declared from personal experience that medicines by the mouth had no effect on this symptom, and recommends morphia hypodermically or by rectum. Nothnagel contested the statement that formed fæces were ever thrown up. He had never seen it, and did not believe it possible. Sometimes formed masses are found in the vomit, which consist of coagulated milk coloured with bile: at the autopsy one never finds formed fæces above the point of obstruction. Leube answered the question, "When may and when must one operate?" by saying that when internal remedies are exhausted, and the pulse still good, we might do so, and it ought certainly to be done when the pulse becomes bad. Rosenbach advocated the intestinal puncture, being supported in this by Fraentzel; but Hoffman of Leipzig held that, even with great care and a very fine trocar, fæcal matter may escape into the abdomen. All the speakers spoke with approval of the washing-out of the stomach recommended by Kussmaul; Mosler even declaring that in many cases it led to cure, in others it relieved, and by lessening the abdominal pressure made palpation much easier for the practitioner, and increased the chances of a correct diagnosis.—*Therapeutische Monatshefte*, Heft 5, 1889, and *Berliner klinische Wochenschrift*, No. 17, 1889. D. R. P.

**Action of Sulphonal.**—This drug, which was introduced by Kast as a trustworthy sleep-producing agent, has now been on its trial for a year; and although in certain cases it has been followed by striking effects, the result of experience seems to indicate that we have not in it a safe and certain hypnotic free from unpleasant after-consequences. The prostration, bodily and mental, which sometimes follows its use, is often very marked, and is well illustrated by the following cases recorded by Rehm:

1. A lady, aged 48, suffering from muscular rheumatism in the neck, was treated by friction and massage in vain; but was much relieved by baths. The sleeplessness was combated by 22-gr. doses of sulphonal, which she took on six consecutive evenings. On the first three occasions it acted agreeably; but after further doses constipation, loss of appetite, restlessness, excitement and anxiety, with illusions and hallucinations, set in. She was in a condition of great exhaustion, very pale, with speech scarcely audible. There was great hyperæsthesia of the senses, double vision, and muscular twitching. She was unable to sit up, the movements of the hands were ataxic, and she could not raise her eyelids. After the discontinuance of sulphonal she recovered slowly; the power of locomotion was much affected even four weeks after, and it was some time before she was quite well again.

2. A gentleman, 51 years of age, was given for sleeplessness 30 grs. sulphonal as a narcotic. All night he felt as the bed were swinging. During the whole of the following day he lay in a very stupid state, had no appetite, and felt ill. He vomited several times, and felt very giddy. Some days after the drug was tried again, but the same effects followed.

3. A nervous lady, 32 years old, for years moderately addicted to the use of morphia, took 30 grs. for sleeplessness. One dose was taken, after which she lay for four days feeling tired, sleepy, stupid, and giddy; it had no influence on digestion.

A curious contrast to these cases is afforded by an instance which occurred in Riedel's chemical manufactory, where a workman, "in order to get a right good sleep," took three good tablespoonfuls of sulphonal. He remained for half-an-hour in a beerhouse, and went home only when he felt overcome by weariness. He slept from Thursday evening to Tuesday morning, and then, after he was awakened, went to sleep again until Wednesday afternoon. He felt somewhat giddy; but although he had taken 450 grs. of the drug, he felt no other unpleasant after-effects.—*Berliner klinische Wochenschrift*, No. 16, 1889. D. R. P.

**Cure of Exophthalmic Goitre by treatment of a Nasal affection.**—Hopmann contributes a third case to this mode of treatment. Early in 1888 Professor Fränkel brought forward an instance where an incomplete case—palpitation and goitre without exophthalmos—was greatly improved by the treatment of a nasal stenosis by the Galvano-cautery. The second case was communicated in 1886 by Hack, where treatment of the hypertrophied inferior turbinated bone by the Galvano-cautery led to the cure of the disease. Hopmann's case was a female, aged 40, who complained of throat-trouble, constant hawking with cough. There was great weakness in the legs, palpitation and pain in the eyes, especially the right. There was exophthalmos in the right eye, with Graefe's symptoms. Here there was no goitre; but Bamberger points out that the disease may occur without one or even two, of the cardinal symptoms. The rhinitis and pharyngitis were treated: two or three small polypi, as well as some crusts, were removed from the nose, with the result that the symptoms soon completely disappeared.—*Berliner klinische Wochenschrift*, No. 42, 1888. D. R. P.

